# Differentiating network structures and sex differences of pain-related outcomes, analgesic opioid dosages, and psychosocial factors for postoperative management: a study of PAIN OUT registry in seven Asian regions

**DOI:** 10.1186/s41256-025-00442-w

**Published:** 2025-10-07

**Authors:** Yulin Huang, Hung Chak Ho, Yuchang Bao, Ruth Zaslansky, Painout ASPIRE Network, Lorina Cabaluna, Kok Yuen Ho, Andrew Ng, Siu Min Lim, Chunlong Chen, Salah El-Tallawy, Jun Yin, Cunming Liu, Xuehua Zhang, Zhiyao Wang, Mingyang Chen, Jiannan Song, Donghua Liu, Chuandong Zheng, Weian Zeng, Qiong Wu, Ying Wang, Jeffrey Ip, Dwi Pantja, Winfried Meißner, Chi Wai Cheung

**Affiliations:** 1 Department of Neuroscience, City University of Hong Kong, Kowloon, Hong Kong, China; 2 Shanghai Key Laboratory of Brain Functional Genomics (Ministry of Education), School of Psychology and Cognitive Science, East China Normal University, Shanghai, China; 3 NYU-ECNU Institute of Brain and Cognitive Science, New York University Shanghai, Shanghai, China; 4 Department of Public and International Affairs, City University of Hong Kong, Kowloon, Hong Kong, China; 5 Social Determinants of Health Initiative, City University of Hong Kong, Kowloon, Hong Kong, China; 6 Department of Anaesthesiology, School of Clinical Medicine, The University of Hong Kong, Hong Kong, China; 7 Department of Anesthesiology and Intensive Care, University Hospital Jena, Jena, Germany; 8 Department of Anaesthesiology, Hong Kong Sanatorium & Hospital, Hong Kong, China

**Keywords:** Postoperative pain, Pain management, Oral morphine equivalents, Network analysis, Psychosocial factors, Sex difference

## Abstract

**Background:**

Despite advancements in global healthcare, Asia lacks a systematic framework for postoperative pain-related symptoms, particularly in addressing pain identification/prevention and reporting sex differences. This study used network analysis to unravel the complex interplay of pain-related symptoms, analgesic opioid dosages, and psychosocial factors, with a particular focus on sex differences in the Asian population.

**Methods:**

Utilizing anonymized data from 5,093 adult patients across seven Asian regions between 2018 and 2021 from the PAIN OUT registry, this study applied network analysis: 1) to map the relationships between analgesic opioid dosages and multidimensional pain-related symptoms and 2) to explore sex differences. This network analysis was performed based on information from the International Pain Outcomes Questionnaire (IPO-Q), which included pain severity, adverse events, perceptions of pain care perception, and pain treatment satisfaction within 24h of an operation. The model utilized Least Absolute Shrinkage and Selection Operator (LASSO) for regularization and edge estimation, a penalized estimation method allowing for the identification of the most relevant connections while effectively controlling for overfitting.

**Results:**

Network structures demonstrated high stability, revealing distinct sex-based patterns. Chronicity of pain (#CP) emerged as the most central node in the overall network structure (*EI* = 1.50) and among male patients (*EI* = 1.80), reflecting the profound effect of persistent pain on their functional activities and sensory-focused symptoms, such as dizziness (#AE4). In contrast, helplessness (#MH2) was a significant symptom in female patients (*EI* = 1.70), highlighting the emotional and psychological dimensions of their pain experience. Comparative analysis uncovered significant structural differences between males and females (M = 0.154, p = 0.023), emphasizing the unique interplay of psychological, emotional, societal, and pathophysiological symptoms in shaping postoperative pain experiences.

**Conclusions:**

This study was the first comprehensive network analysis of pain-related symptoms with sex differences. The results highlighted a significant difference in associations between analgesic opioid dosages and multidimensional pain-related symptoms among males and females, implying the necessity for region-specific, multimodal interventions to optimize postoperative care in Asian populations.

**Supplementary Information:**

The online version contains supplementary material available at 10.1186/s41256-025-00442-w.

## Introduction

Postoperative pain, as a common surgical complication, represents a major challenge to the healthcare. According to the clinical practice guidelines from the American Pain Society, effective management of postoperative pain requires a multi-modal approach, utilizing both pharmacological and non-pharmacological interventions [1]. This approach, known as multi-modal analgesia, is essential for optimal pain relief [1]. The International Association for the Study of Pain (IASP) defines pain as an unpleasant sensory and emotional experience associated with tissue damage [2]. Preoperative anxiety, often driven by the anticipation of postoperative pain, is common among patients [3], highlighting the importance of healthcare providers to deliver professional care and the implement effective strategies to manage acute postoperative pain, while addressing the concerns and fears of patients.

Effective postoperative pain management is not only crucial for alleviating discomfort but also plays a role in supporting postoperative recovery, minimizing complications, and improving overall clinical care. Considering cultural perceptions and attitudes towards pain, it is essential to understand local contexts when conducting postoperative pain management [4, 5]. This is especially pertinent in Asian patients. Despite significant variations in economic development and medical resources [6], Asian countries exhibit similar cultural attitudes and nursing practices towards postoperative pain management.

Culture factors in Asian often promote the suppression and tolerance of pain, which can lead to an underestimation of pain levels in postoperative assessments[7, 8, 8]. Furthermore, resource allocation challenges of healthcare systems, such as limited medical resources, particularly in rural or economically underdeveloped areas[9], may result in suboptimal postoperative pain treatment[10]. Given these characteristics, it is important to develop and implement postoperative pain management’s strategies tailored to the specific needs of Asian populations.

Effectively managing postoperative pain requires multi-modal approaches, as previously mentioned [1, 11]. The biopsychosocial model of pain offers a comprehensive framework for understanding and treating pain, recognizing that pain is not solely a physiological phenomenon but can also be influenced by psychological and sociocultural factors [12]. The “neuromatrix” theory further emphasizes the multi-dimension nature of pain, proposing that the pain matrix (including brain regions) can be responsible for not only active pain perception but also emotional and cognitive processes [13]. These models integrate biological, psychological, and social factors, providing a solid theoretical foundation for understanding and managing postoperative pain. Exploring the intrinsic dynamic structure of these factors is crucial for developing more comprehensive and effective postoperative pain management’s strategies.

Postoperative pain remains a significant surgical complication in healthcare, necessitating its effective pain management in healthcare, particularly in Asia, where comprehensive research on its multidimensional characteristics is limited. Traditional postoperative pain studies typically used regression and cluster analysis [14, 15], but these methods had limited abilities to elucidate bidirectional relationships and complex interactions between variables. We performed a literature search on PubMed and Web of Science using the following keywords and their combinations: "postoperative pain," "pain management," "Oral Morphine Equivalents (OMEs)," and "network analysis." Although numerous studies on sex differences in postoperative pain outcomes have highlighted the potential roles of biological factors in pain perception and recovery, these studies often overlooked the complex interrelationships among various postoperative pain outcomes, including pain severity, mental health, and analgesic opioid dosages. Furthermore, limited comprehensive and systemic analyses on postoperative pain management in Asia hinders a thorough understanding of the multidimensional characteristics of postoperative pain in this population.

Therefore, the current study employed a network analysis to address the above limitations. In the field of pathology, nodes typically represent symptoms (i.e., variable), while edges signify the connections between nodes [16]. The thickness of the edges can be understood as a measure of the likelihood of co-activation [16]. By employing network analysis, postoperative pain, a common surgical complication, is conceptualized as a complex system characterized by dynamic interactions between multiple symptoms and conditions.

The aim of this study was to employ network neuroscience principles through Gaussian graphical modeling to explore the pathological and clinical significances of the topological structure of acute postoperative pain experiences in Asian populations, focusing on identifying sex-based nodal characteristics. Using prospectively collected data from the PAIN OUT registry, which spans over 25 tertiary hospitals across seven Asian regions, we constructed multidimensional pain networks encompassing physiological, psychological, and functional domains. Nodal centrality was quantified using expected influence metrics to identify potential intervention targets, and comparative network analyses were conducted to examine differences between males and females.

## Methods

### Data sources

This study was conducted using anonymous data from PAIN OUT ASPIRE operated by PAIN OUT registry. PAIN OUT registry is an international collaborative project to evaluate and analyze the outcomes of postoperative pain management (https://www.pain-out.eu/drupal/painout/). The PAIN OUT ASPIRE project (POAP) was conducted from 2018 to 2021 and covered more than 25 tertiary hospitals in Indonesia, Singapore, Hong Kong Special Administrative Region (China), Malaysia, Saudi Arabia, the Philippines, and China. The main goal of this project was to collect anonymous feedback of patients on their satisfaction with pain treatment and to improve pain management. Specifically, trained evaluators collected anonymous demographic information via questionnaire preoperatively, including sex, age, nationality, and preoperative use of analgesic medications. During the perioperative period, details of sedative medication and analgesic opioid dosage were recorded. Finally, participants were asked to report postoperative pain intensity, pain-related functional impairment, adverse effects of pain treatment, and satisfaction with pain management within the first 24 h. The use of anonymous data for analysis in this study was approved by the Human and Artefacts Ethics Sub-Committee, City University of Hong Kong (Code: HU-STA-00001478).

### Study design and subjects

**Fig. 1:**
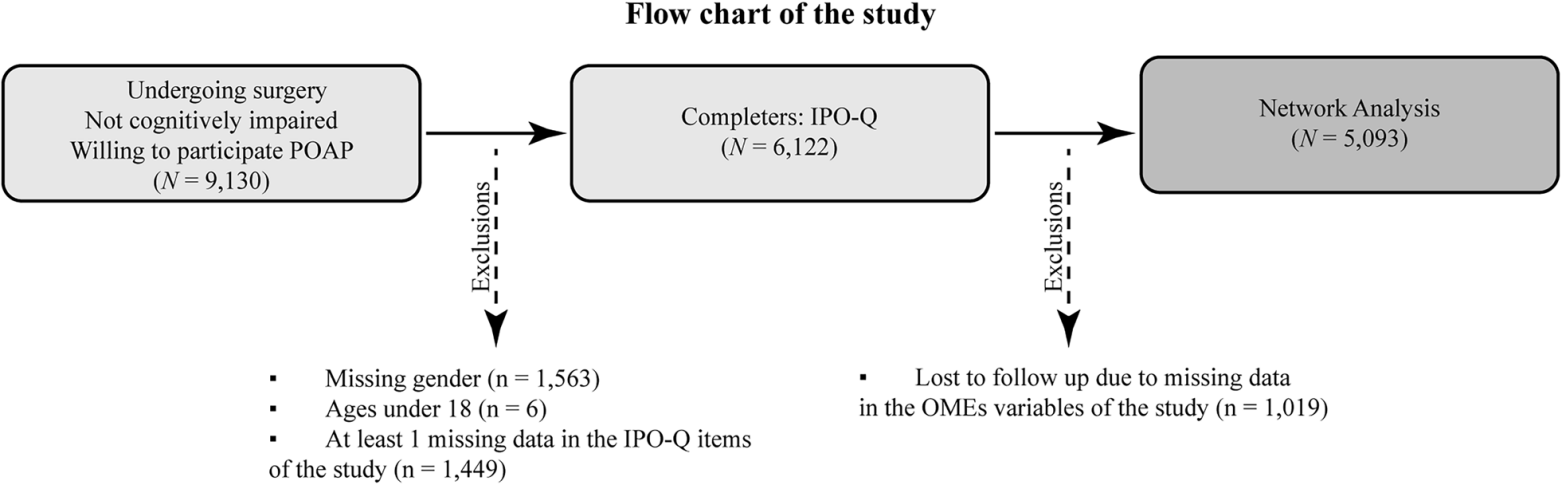
Flow chart of the study. IPO: International Pain Outcome Questionnaire; OMEs: Oral morphine equivalents

This study applied a cross-sectional design. A total of 9,130 patients were initially recorded in POAP. The inclusion criteria for the POAP study were as follows: patients who 1) underwent intraoperative procedures; 2) aged 18 years or above; 3) did not with cognitive impairment; and 4) provided consent to participate in a survey to assess perioperative pain-related outcomes. Patients with missing or "unrated" evaluation in pain-related ratings and feedback, as well as missing information on analgesic opioid dosage, were excluded from the current analysis. A flowchart was constructed based on the inclusion and exclusion criteria outlined for the database (Fig. 1). The final sample consisted of 5093 participants who met entry requirements, of which 2882 were women (55.61%) (Mean_age_ = 56.76, SD_age_ = 15.65).

### Variables

Pain-related outcomes. Preoperative pain-related outcome was assessed by chronicity of pain (#CP), a self-reported variable of chronic pain. Postoperative pain-related outcomes were assessed using the International Pain Outcomes Questionnaire (IPO-Q) [17]. The questionnaire consists of three outcome domains including (1) pain intensity and interference: worst pain (#PI1), least pain (#PI2), severe pain (#PI3), in-bed activities (#IF1), breathing and coughing (#IF2), sleep and out-bed activities (#IF3), anxiety (#MH1) and helplessness (#MH2); (2) adverse effects: nausea (#AE1), drowsiness (#AE2), itching (#AE3) and dizziness (#AE4); and (3) perception of care: pain relief (#PC1), treatment decisions (#PC2), satisfaction (#PC3). All variables except “Out of the bed”, “Severe pain” and “Pain relief” were rated using an 11-point Numerical Rating Scale (NRS). The item “Out of the bed” was evaluated with a dichotomous yes/no scale, while “Severe pain” and “Pain relief” were measured with a percentage scale from 0 to 100%. In this study, considering that the majority of patients were unable to walk in the postoperative period, assessment of pain interference with out-of-bed activities was excluded from the analysis. However, the influence of pain on the ability to get out of bed was considered. The psychometric properties of the IPO-Q have been validated in English and subsequently translated into 29 languages using a standardized methodology [17].

Oral morphine equivalents (OMEs) for analgesic opioids. The current analysis included dosage of analgesic opioids administered in four phases: preoperative period (#ME1), intraoperative period (#ME2), recovery room (#ME3), and ward (#ME4). These dosages were converted to oral morphine equivalents (OMEs) using published equianalgesic tables (Table. S5) [18]. Oral analgesic opioids were converted directly to OMEs, while intravenous opioids were first converted to intravenous morphine and then adjusted by a bioavailability factor of 3 to determine OMEs [19].

### Statistical analysis

Previous evidences showed that age, sedative use, non-opioid medications, and physical therapy may affect postoperative pain [11, 15]. Consequently, we controlled for these variables and subsequently estimated the network model and local structure indices via using the Mixed Graphical Model (MGM)[19]. To estimate the MGM network, we employed the Least Absolute Shrinkage and Selection Operator (LASSO) for regularization and cross validation (CV) for the selection of optimal regularization parameters [19]. This approach can mitigate overfitting and result in a sparse network structure, enhancing the interpretability compared to the original network [20].

Each item of the IPO-Q and OMEs at different perioperative stages was treated as a “node,” and the connections between these nodes were treated as “edges”. The current study primarily focused on significant edges that 1) can have strong association across different domains of IPO-Q outcomes and 2) can induce a high association in overall network structure. The expected influence (*EI*) metric was used to assess node centrality by summing the weights of all directly connected edges, which can be extended to include indirect connections [23]. To ensure the robustness of the network model and to address the potential uneven distribution of surgical types and urban–rural disparities across the seven regions in our dataset, we 1) implemented a random resampling procedure and 2) applied a dual approach to evaluate the accuracy and stability of the network model. Specifically, we first utilized the *bootnet *package to perform 10,000 bootstrap resamples, calculating the Correlation Stability coefficient (*CS-C*) to assess the stability of the centrality metrics (i.e., *EI*) [24]. The *CS-C* represents the maximum proportion of the sample that can be removed while ensuring the stability of the centrality metric, derived through subset bootstrap methods [21]. A *CS-C* above 0.25, and preferably above 0.5, indicates a stable metric, as proposed by Epskamp et al.[21]. These thresholds are widely used in network analysis research [25–27], and are supported by simulation studies demonstrating that a *CS-C* above 0.5 provides sufficient stability for centrality indices in networks constructed from similar sample sizes and data structures [21]. Secondly, we calculated the 95% non-parametric confidence intervals (*CIs*) for the edge weights through 10,000 bootstrap resamples to evaluate the accuracy of the edges [21]. Larger *CIs* indicate lower precision in the edge estimates, whereas smaller *CIs* suggest higher reliability of the network.

Male and female patients were then compared on the items-level scores of the IPO-Q. Based on previous research [28, 29], we focused on the proportions of patients with moderate to long phase levels (≥ 4). The R package *NetworkComparisonTest* (NCT) was used to examine the differences in network characteristics between male and female patients [30]. NCT is permutation test used to examine the invariance of different network characteristics. In this analysis, we compared networks of male and female patients using the NCT function with 10,000 iterations. The Bonferroni method was applied to adjust p-values for testing sex differences in global *EI*.

Graphic visualization was used to demonstrate the association and weight of each edge. In this study, green edges on the graphic visualization represented positive association, while red edges indicated negative association. The thickness of the edges indicated a measure of the likelihood of co-activation. The thicker the edge, the bigger the weight.

All statistical analyses were conducted in R. For the estimation and graphic visualization of the network, the R packages *bootnet*[21], *networktools*[22], and *qgraph*[23], were used.

## Results

### Descriptive analysis

Table 1 summarizes the descriptive characteristics of the variables used in the network analysis. Among 5,093 selected participants, 22.46% used sedatives preoperatively, 94.13% used non-opioid analgesics, and 33.34% employed pain relief’s methods without medicine. Chronic pain conditions were reported by 30.63% of patients. Perception for pain relief was high, (Mean_PC1_ = 7.20; SD_PC1_ = 2.46), with an overall satisfaction for pain management (Mean_PC3_ = 8.26; SD_PC3_ = 1.97). More details regarding IPO-Q and OMEs nodes can be found in Table 1 and the following sections.

**Table 1:**
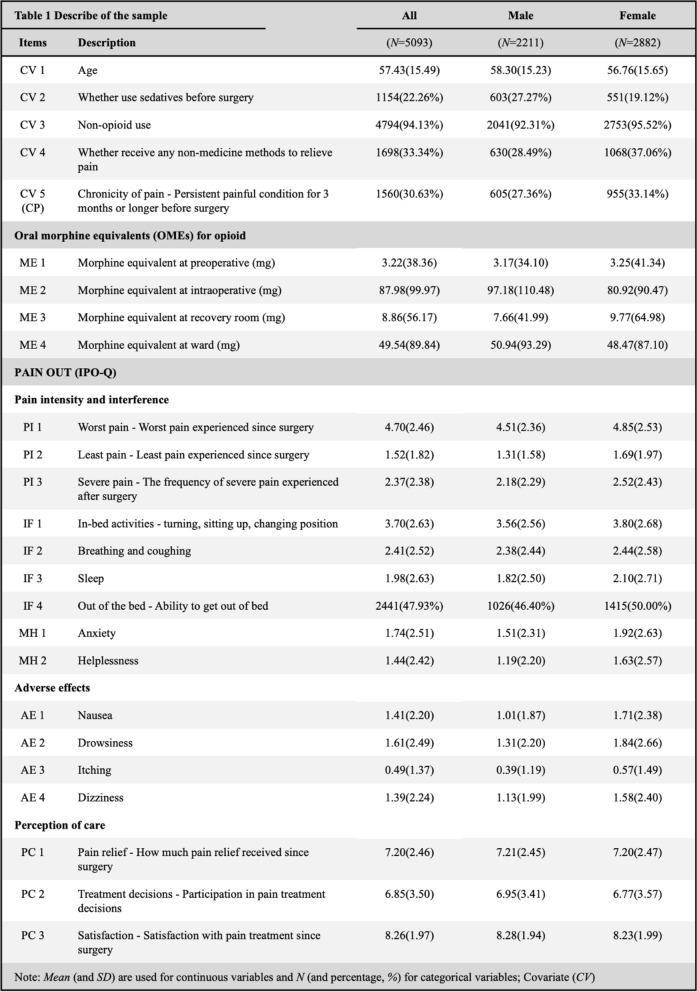
Descriptive summary of all patients included in this study (*N* = 5093)

### Network estimation and analysis of centrality measure

**Fig. 2:**
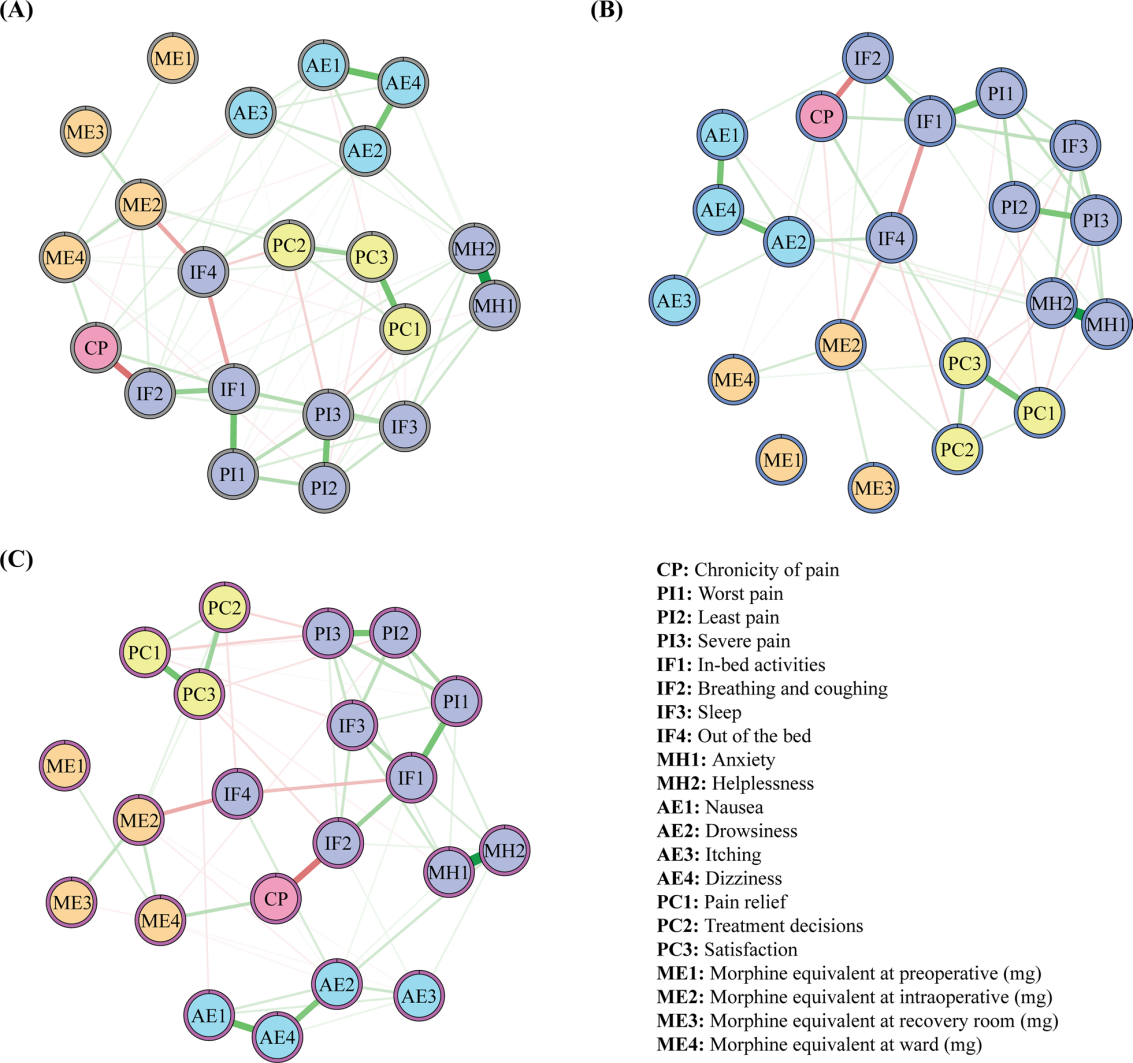
Visualizations of network structure. Figure 2A indicates the network structure of all patients. Figure 2B indicates the network structure of male patients. Figure 2C indicates the network structure of female patients. Nodes in purple indicate factors related to pain intensity and interference. Nodes in blue are factors related to adverse effects. Nodes in yellow indicate factors related to perception of care. Nodes in orange are morphine equivalent usage. Node in pink is chronicity of pain. Additionally, green edges indicate positive association and red edges indicate negative association. Higher thickness of an edge reflects a stronger association between variables

**Fig. 3:**
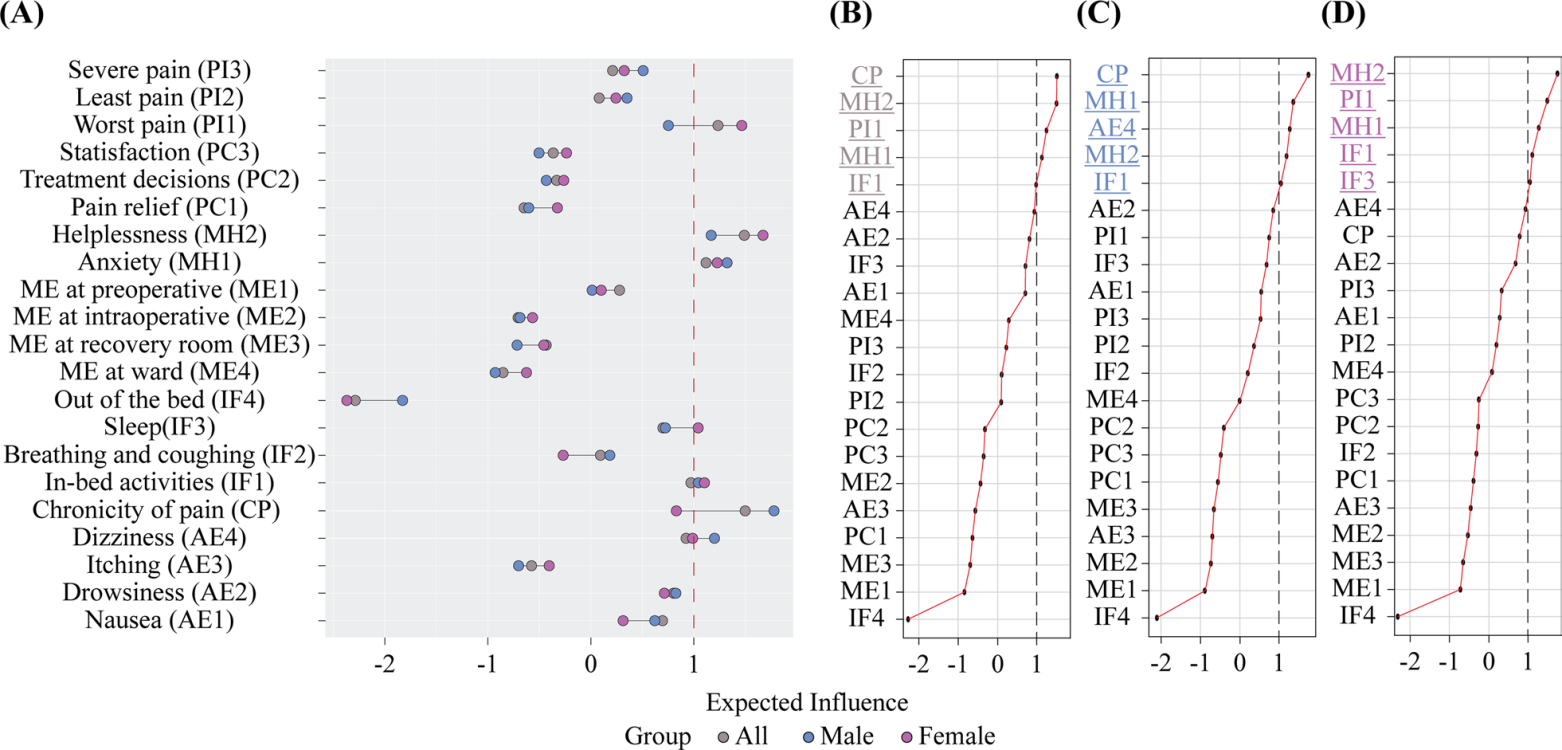
The expected influence (EI) of variables from network analyses. Figure 3A indicates the comparisons of EI for three groups (all, males and females). The X-axis represents the EI values (the relative centrality of each variable in the network), which measure the extent to which each factor is directly or indirectly connected to others in the network. Figure 3B to 3D indicates orders of EI for all, males, and females. The order of the variables on the Y-axis is arranged according to their centrality ranking, with the most influential variables (highest EI) appearing at the top and the least influential variables (lowest EI) at the bottom. Specifically, variables with grey color in Fig. 3B indicate the most influential variables for all participants, variables with blue color in Fig. 3C indicate the most influential variables for males, and variables with purple color in Fig. 3D indicate the most influential variables for females

The network structure for all patients is presented in Fig. 2A. The network structure comprised 25 nodes and 300 edges, with 108 non-zero edges (36.00%) identified. In the network of all patients (Fig. 3B; Table S4), #CP (chronicity of pain, *EI* = 1.50) exhibited the highest *EI*, followed by #MH2 (helplessness, *EI* = 1.49), #PI1 (worst pain, *EI* = 1.23), and #MH1 (anxiety, *EI* = 1.12). Several highly associated edges were identified in the network of all patients (Table S1), including #MH1 (anxiety)—#MH2 (helplessness) (*r* = 0.596), #CP (chronicity of pain)—#IF2 (breathing and coughing) (*r* = -0.335), and #IF1 (in-bed activities)—#PI1 (worst pain) (*r* = 0.335).

The network structures for male and female patients are presented in Fig. 2B and 2C. Both networks comprised 25 nodes and 300 edges, with 79 non-zero edges (26.33%) identified in the network of male patients and 78 non-zero edges (26.00%) in the network of female patients. The most central node of the network of male patients was #CP (chronicity of pain, *EI* = 1.78), followed by #MH1 (anxiety, *EI* = 1.32), #AE4 (dizziness, *EI* = 1.20) and #MH2 (helplessness, *EI* = 1.17) (Fig. 3C; Table S4). The highest *EI* in the network of female patients were observed in #MH2 (helplessness, *EI* = 1.67), followed by #PI1 (worst pain, *EI* = 1.47), #MH1 (anxiety, *EI* = 1.23), and #IF1 (in-bed activities, *EI* = 1.10) (Fig. 3D; Table S4). Notably, the three most prominent edges in the network of all patients also exhibited strong association in both the male and female’s networks (Tables S1, S2, S3), including #MH1 (anxiety)—#MH2 (helplessness) (*r* = 0.592 ~ 0.595), #CP (chronicity of pain)—#IF2 (breathing and coughing) (*r* = -0.291 ~ -0.321), and #IF1 (in-bed activities)—#PI1 (worst pain) (*r* = 0.319 ~ 0.345).

The majority of edges associated with OMEs across the three networks exhibited weight below 0.10 in the current sparse network. Many edge weights associated with OMEs were reduced to an approximate zero after the application of LASSO regularization, indicating no association between nodes. Among these, #ME2 (OMEs at intraoperative)—#IF4 (out of the bed) (*r* = -0.157 ~ -0.207) and #CP (chronicity of pain)—#ME4 (OMEs in ward) (r = 0.033 ~ 0.156) exhibited a relatively prominent negative association in the domain of OMEs outcomes.

Furthermore, our results also showed that the perception of care (#PC1, #PC2 and #PC3) had weak edge weights (r ≤ 0.10) with other pain-related nodes within the network. In the network of all patients, #PI3 (severe pain) and #IF4 (out of the bed) had relatively prominent negative associations with #PC2 (treatment decision), but these *r* remained low, ranging only from -0.101 to -0.105.

### Network comparison between male and female patients

**Fig. 4:**
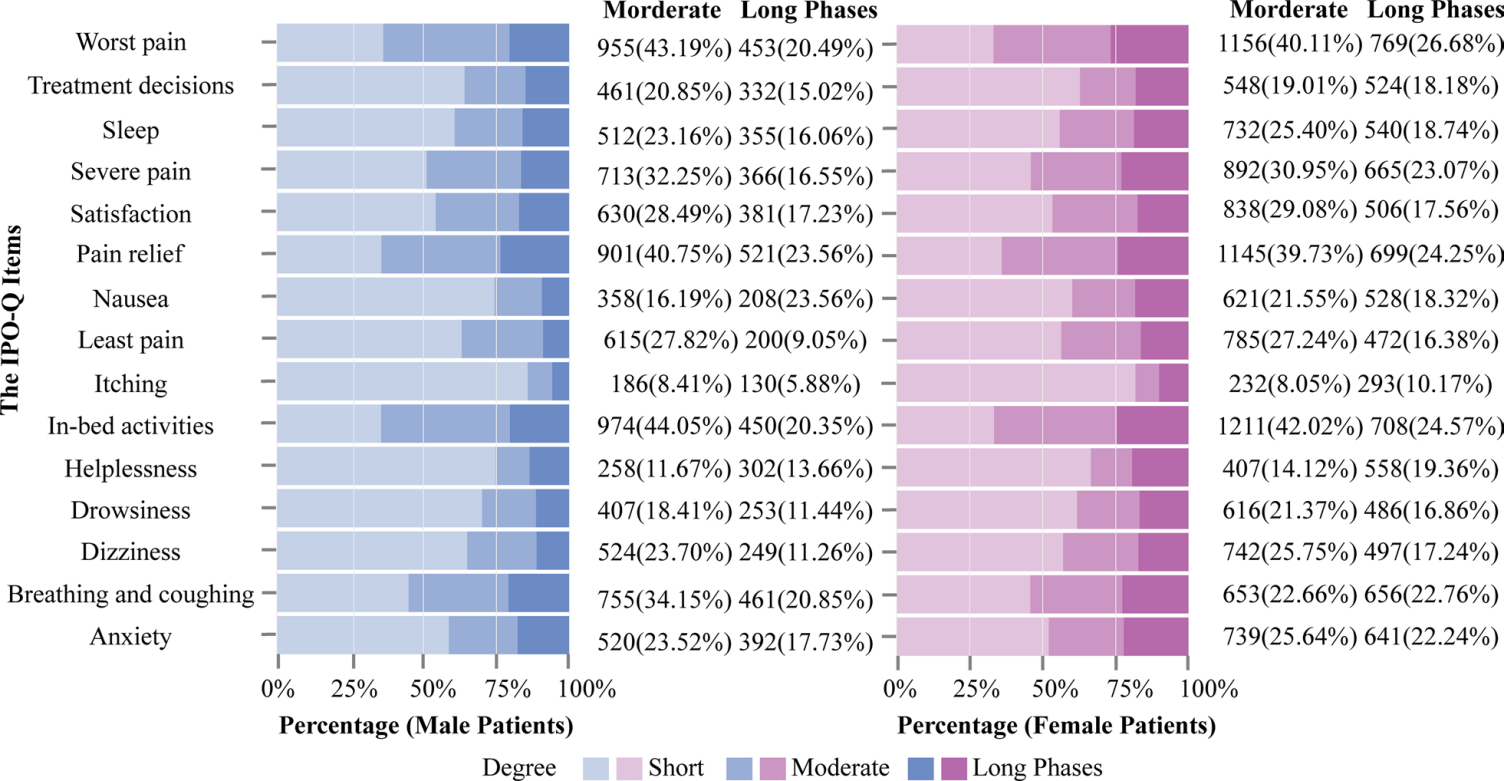
Network comparison between male and female patients. Figures indicates the prevalence of mild, moderate, and long phases acute postoperative pain in male patients (left/blue color) and female patients (right/purple color)

The distribution between male and female patients was similar (Fig. 4), with an effect size of 0.339. Female patients had slightly higher proportions of moderate to long phases than male patients in most IPO-Q variables, including worst pain (66.79%) and helplessness (33.48%).

We compared the network models and network centrality indices (i.e., *EI*) of male (*N* = 2211) and female (*N* = 2882) patients via the NCT. While there were overall significant structural differences between the networks of male and female patients (*M* = 0.154, *p* = 0.023), these differences did not result in significant changes in global expected influence for either group (*S* = 0.038), with a *p*-value of 0.896. This implied that although certain connections or edge weights within the network structure may exhibit significant changes, these alterations did not significantly affect the influence of nodes as central EI points within the network (all *p* > 0.05 after Holm–Bonferroni corrections).

### Network accuracy and stability

We performed a bootstrapped analysis to ensure the stability of all network models. The results showed that the 95% CIs were narrow in the three networks (all patients, male patients, and female patients) (Fig. S1). The narrow CIs in these figures indicated that the network structure was accurately represented. Furthermore, the case-dropping bootstrap procedure demonstrated that the *CS-C* were uniformly 0.75 in the network of all and female patients (Fig. S2), and the *CS-C* in the network of male patients was 0.62. These results indicated excellent stability. Consequently, the network model and the centrality metrics (i.e., *EI*) can be deemed robust and interpretable based on these stability indicators. Data can exhibit acceptable stability even when the sample was randomly resampled, even with the sample size reduced to less than 30%.

## Discussion

The current study addressed the aforementioned gap in postoperative pain research by employing network analysis to examine postoperative pain outcomes in seven Asian regions between 2018 and 2021 using data collected from the Aspire Project. To our knowledge, this was the first study to use network analysis to examine postoperative pain management among male and female patients in Asia. Our data included patients from various tertiary hospitals across several Asian countries. For male patients and the overall population, “chronicity of pain” (#CP) emerged as the most central symptom, while in female patients, “helplessness” (#MH2) was the most prominent. These findings revealed intrinsic dynamic interconnections between postoperative pain and factors such as emotional states, medical history, and perioperative medication. These results can offer valuable insights for improving postoperative care in Asian tertiary hospitals.

### Key findings and interpretation

Advanced from previous studies, we utilized a network analysis 1) to overcome the limitations of conventional statistical methods, and 2) to identify key nodes and pathways that may drive postoperative pain dynamics. Network analysis can quantify 1) edge weights to describe symptom-symptom interactions and 2) centrality metrics to identify core drivers of pain networks across males and females. By focusing on sex differences, this study provided unprecedented insights into the unique psychological, emotional, societal, and pathophysiological dimensions of pain. These findings not only addressed a critical knowledge gap but were also able to propose various strategies for innovative, evidence-based pain management that can tailor to the needs of diverse populations.

Another key finding of this study was the significant sex differences in the network structure of postoperative pain. Biological differences in how the central nervous system processes pain signals likely contributes to these variations [36]. Specifically, women may experience amplified pain signals [37, 38], resulting in more intense pain experiences (#PI1), while men tended to report a greater impact of pain on functional activities [39, 40], particularly sensory-focused symptoms such as dizziness (#AE4). Additionally, women may use certain adaptive mechanisms in response to the chronicity of pain (#CP), such as heightened pain sensitivity and an enhanced ability to distinguish different levels of pain [41]. Our analysis suggested that chronic pain had a greater impact on men compared to women. Differences in pain perception during the menstrual cycle may provide women with more complex and diverse coping mechanisms for managing chronic pain.

The above results implied that traditional gender roles may also play a role in how men and women cope with pain. Women are more likely to use emotion-focused coping strategies, such as seeking social support and expressing emotions [40, 42]. However, these strategies may lead to feelings of helplessness when pain becomes unmanageable (#MH2). This consistent with the theory of learned helplessness, where individuals perceive lack of control and problem-solving ability despite their efforts [43]. In contrast, men often adopt problem-focused coping strategies [42], and when these fail, they may feel anxiety and tension [44]. Additionally, societal expectations often pressure men to display self-reliance and control [45]. Failure to do so may be unable to cope with their pain and increase their feelings of depression and anxiety (#MH1) [44].

Our results also implied that physical activity may play a significant role in modulating pain and mental health. The release of endorphins during physical activity acts as a natural painkiller and mood enhancer [46]. However, our findings suggested that severe pain may limit a person’s ability to engage in activities in bed (#PI1—#IF1). When physical activity was restricted, endorphin levels may decrease, exacerbating feelings of depression, helplessness, and anxiety [46]. Given these sex-specific coping strategies and the neurobiological effects of physical activity on mental health, it is not surprising that anxiety and helplessness exhibited a strong connection (#MH1—#MH2) in our study [47]. The robust link between these emotional states suggested that these mental distresses may frequently co-occur and influence each other. Both mental distresses are commonly associated with imbalances in neurotransmitters, such as serotonin, norepinephrine, and dopamine [48, 49]. Additionally, dysregulation of the hypothalamic–pituitary–adrenal (HPA) axis has also been implicated in the development of anxiety and helplessness [50, 51].

As “chronicity of pain” was the most central symptom for males and the overall population identified in this study, its potential impacts should be noted. In this study, chronicity of pain was closely linked to respiratory symptoms, such as breathing and coughing (#CP—#IF2). Experiences of chronic pain can significantly alter respiratory patterns, causing shallow and rapid breathing [52]. Patients may adjust their breathing patterns to alleviate pain [52]; however, such changes can result in persistent muscle tension and stress, thereby exacerbating cough. Furthermore, patients with experiences of chronic pain may also require higher dosage of medication during ward (#CP—#ME4) due to the development of drug tolerance, which necessitates careful management to avoid under-treatment of pain [53, 54].

Several specific evidences observed in this study should also be noted.

1. It is commonly believed that analgesic opioids are essential for controlling postoperative pain. However, the current results showed that analgesic opioid dosage was a comparatively less influential factor, compared with other factors within the pain-related network. This finding may be attributed to multiple factors, such as potential underdosing due to 1) concerns over opioid misuse or side effects, 2) development of opioid tolerance over time, 3) unmeasured confounding factors (e.g., BMI, comorbidities), and 4) genetic variations in opioid metabolism. These factors may lead to an unproportioned analgesic prescriptions and/or dosage that were able to meet the needs of pain management among patients [55]. The interactions between opioids and other non-pharmacological or adjunctive treatments, as well as sociocultural factors influencing pain perception and related outcomes may also play a role. Specifically, postoperative pain and medication use are closely related to a detailed medical history. For instance, obesity-related physiological changes can influence drug absorption, distribution, metabolism, and clearance [56, 57]. Moreover, severe comorbidities, such as diabetes, or other chronic conditions like respiratory or hepatic dysfunction, may influence opioid metabolism, distribution, and clearance, potentially altering their efficacy, as well as impacting the patient’s pain perception and tolerance [57, 58]. The specific mechanisms underlying these observations are required for further investigation.
2. The prevalence of moderate to long phases postoperative pain in our data was significantly lower than that in the United States (75%) and Ethiopia (88.2%) [4, 5]. The above results suggested that pain experiences may vary across populations, with potential influences from local conditions. The profound effects of cultural beliefs and values on pain perception and expression, as highlighted in previous cross-cultural studies [31, 32], can be one of the potential influential factors. Cultural attitudes in many Asian societies usually emphasize pain tolerance and endurance as virtues. These attitudes may lead to an underreporting or a normalization of pain experiences that can reduce willingness to seek medical assistance [31]. Differences in allocating health resources may be another influential factor. According to the World Health Organization (WHO)’s Global Health Expenditure Database, Western countries allocate significantly more resources to healthcare, enabling advanced medical technologies and comprehensive care [33]. In contrast, some Asian regions, even in tertiary care centers, often experience resource constraints, which may limit access to effective pain management and impact patient outcomes [34].
3. Our findings aligned with previous studies that pain perception may significantly influence postoperative physical activity, sleep, emotions, and the adverse effects of pain treatment [9, 59, 60]. However, our findings did not show a strong association between patient satisfaction and postoperative outcomes [9]. This discrepancy could be partly explained by expectations regarding care. Patient satisfaction may be more closely linked to the physician’s communication style, the level of understanding of the patient, and empathy, rather than tangible clinical outcomes such as pain intensity and tolerance [35]. Our data covered multiple tertiary hospitals across Asia regions, and their procedures for healthcare and support were varied. This may affect how pain management translated into substantial improvement in pain or postoperative recovery, which resulted in a less significant association between patient satisfaction and postoperative outcomes. Furthermore, measurement biases in how patients reported satisfaction, recovery process and outcomes may also contribute to this inconsistency.

### Implications for clinical research

Our findings highlighted the necessity of research on postoperative pain across diverse populations, whose pain tolerance and experiences may differ significantly. Notably, our study revealed a lack of association between pain alleviation and patient satisfaction, as well as insufficient evidence supporting opioid analgesics efficacy. Moreover, the significant differences in network structures between males and females highlighted the importance of sex-based interventions. Chronicity of pain emerged as a central node in males, whereas helplessness was a significantly node in females, reflecting distinct pain processing mechanisms. These insights can call for a paradigm shift in postoperative pain management, emphasizing the development of multimodal, tailored strategies that address the unique needs of patients.

Based on our results, we identified significant network structure differences between male and female patients, as well as the central indicators of acute postoperative pain. While centrality indicators may not always directly equate to clinical relevance, they still can offer valuable guidance, as these indicators may represent the causal endpoints of numerous network pathways. Consequently, we can tailor clinical interventions for male and female patients according to different emphases, with the aims of minimizing pain and promoting recovery. Future research should include more detailed information of patient, such as previous duration of analgesic use, daily lifestyle habits, and social support, as part of the auxiliary analysis to better formulate clinical plans and recommendations. To enhance the clinical significance of the research, future studies should also conduct longitudinal studies to investigate pain progression at different postoperative stages (e.g., within 48 h) and examine differences in pain management in various types of surgeries.

### Limitations

This is a cross-sectional study. Cross-sectional design precludes the establishment of causal relationships. Our findings may not be generalizable to all Asian populations, particularly to those in non-tertiary hospitals or non-surgical contexts, due to differences in healthcare infrastructure or clinical practices. BMI and comorbidities were not included in the study due to a lack of height measurements and detailed comorbidity data. Therefore, our results may not fully reflect the condition of patients with different body types and severe comorbidities. Patients using medications for which OMEs conversions have not yet been researched were excluded from our analysis, potentially limiting the generalizability of our findings regarding medication effects. As a result, resampling and bootstrapping procedures were applied to mitigate the above limitations and resolve issues from unmeasured confounders (e.g., urban–rural disparities of medical resource, surgical type distribution). However, these procedures may not be able to entirely eliminate the impact of selection bias inherent in the dataset. Further longitudinal or interventional studies are needed to confirm the observed associations and explore potential causal pathways. For the purpose of this study, our findings were still relevant as these results were primarily applicable to inpatient surgical populations in tertiary hospitals across Asia.

## Conclusions

The current study employed a network analysis to examine postoperative pain in patients of Asian background. The results showed that postoperative pain is intricately linked with emotional states, chronic pain history, and perioperative medication. Consequently, variables with the highest EI within the network were identified as the preferred potential therapeutic target. The study also revealed that satisfaction assessment’s criteria for Asian patients may not have a significant association with postoperative pain management outcomes, and the dosage of opioid analgesics may not adequately match patients’ pain management needs. Furthermore, sex differences were observed in the structure of pain-related network. Female patients were more likely to experience feelings of helplessness (#MH2) and worst pain (#PI1), whereas male patients were more concerned with the chronicity of pain (#CP) and anxiety (#MH1). Overall, future studies should focus on developing multi-modal intervention tailored to the characteristics and sex differences of the Asian population. Such targeted strategies are essential to enhance the effectiveness of postoperative pain management.

## Supplementary Information

Supplementary file1.

## Data Availability

The datasets generated and/or analyzed during the current study are not publicly available but may be available on reasonable request. More information can be referred to https://www.pain-out.eu/.

## Code availability

Code can be available on reasonable request.
