# Supplementary material for: Differentiating network structures and sex differences of pain-related outcomes, analgesic opioid dosages, and psychosocial factors for postoperative management: a study of PAIN OUT registry in seven Asian regions
Source: Glob Health Res Policy. 2025 Oct 7;10:51. doi: 10.1186/s41256-025-00442-w (PMC12502417; doi:10.1186/s41256-025-00442-w)
Supplement: Supplementary file 1 — Supplementary file1. [file 41256_2025_442_MOESM1_ESM.docx]

Appendices

|  | **CP** | **PI1** | **PI2** | **PI3** | **IF1** | **IF2** | **IF3** | **IF4** | **MH1** | **MH2** | **AE1** | **AE2** | **AE3** | **AE4** | **PC1** | **PC2** | **PC3** | **ME1** | **ME2** | **ME3** | **ME4** |
| --- | --- | --- | --- | --- | --- | --- | --- | --- | --- | --- | --- | --- | --- | --- | --- | --- | --- | --- | --- | --- | --- |
| **CP** | 0 | 0 | 0 | 0 | 0.124 | -0.335 | 0 | 0.041 | 0 | 0 | 0.067 | 0.048 | 0 | 0 | 0 | 0 | 0 | 0 | -0.045 | 0 | 0.12 |
| **PI1** | 0 | 0 | 0.178 | 0.156 | 0.335 | 0 | 0.089 | 0 | 0.044 | 0 | 0 | 0.035 | 0 | 0 | -0.035 | 0 | -0.029 | 0 | 0 | 0 | 0 |
| **PI2** | 0 | 0.178 | 0 | 0.32 | 0 | 0 | 0.118 | 0 | 0.023 | 0.059 | 0 | 0.02 | 0.022 | 0 | -0.05 | 0 | -0.054 | 0 | 0 | 0 | -0.049 |
| **PI3** | 0 | 0.156 | 0.32 | 0 | 0.022 | 0.067 | 0.146 | 0 | 0.1 | 0 | 0 | 0 | 0 | 0 | -0.09 | -0.101 | 0 | 0 | 0 | 0 | 0 |
| **IF1** | 0.124 | 0.335 | 0 | 0.022 | 0 | 0.25 | 0.176 | -0.212 | 0 | 0.077 | 0 | 0.04 | 0 | 0 | 0 | 0 | 0 | 0 | 0 | 0 | 0.032 |
| **IF2** | -0.335 | 0 | 0 | 0.067 | 0.25 | 0 | 0.068 | 0 | 0 | 0.038 | 0.047 | 0 | 0.055 | 0 | 0 | 0.031 | -0.036 | 0 | 0.072 | 0 | 0 |
| **IF3** | 0 | 0.089 | 0.118 | 0.146 | 0.176 | 0.068 | 0 | 0 | 0.105 | 0.105 | 0 | 0 | 0 | 0 | -0.039 | 0 | -0.04 | 0 | 0 | 0 | 0 |
| **IF4** | 0.041 | 0 | 0 | 0 | -0.212 | 0 | 0 | 0 | 0 | 0 | 0 | 0.116 | 0 | 0 | -0.035 | -0.105 | 0.061 | 0 | -0.207 | 0 | 0.043 |
| **MH1** | 0 | 0.044 | 0.023 | 0.1 | 0 | 0 | 0.105 | 0 | 0 | 0.596 | 0 | 0.028 | 0 | 0.046 | -0.034 | 0 | 0 | 0 | 0 | 0 | 0 |
| **MH2** | 0 | 0 | 0.059 | 0 | 0.077 | 0.038 | 0.105 | 0 | 0.596 | 0 | 0 | 0.087 | 0.06 | 0.045 | 0 | 0 | 0 | 0 | 0 | 0 | 0 |
| **AE1** | 0.067 | 0 | 0 | 0 | 0 | 0.047 | 0 | 0 | 0 | 0 | 0 | 0.116 | 0.073 | 0.355 | 0 | 0 | -0.056 | 0 | 0 | 0 | 0.037 |
| **AE2** | 0.048 | 0.035 | 0.02 | 0 | 0.04 | 0 | 0 | 0.116 | 0.028 | 0.087 | 0.116 | 0 | 0.108 | 0.315 | 0 | 0 | 0 | 0 | 0 | 0 | -0.033 |
| **AE3** | 0 | 0 | 0.022 | 0 | 0 | 0.055 | 0 | 0 | 0 | 0.06 | 0.073 | 0.108 | 0 | 0.08 | 0 | 0 | 0 | 0 | -0.031 | 0 | -0.025 |
| **AE4** | 0 | 0 | 0 | 0 | 0 | 0 | 0 | 0 | 0.046 | 0.045 | 0.355 | 0.315 | 0.08 | 0 | 0 | -0.016 | 0 | 0 | 0 | 0 | 0 |
| **PC1** | 0 | -0.035 | -0.05 | -0.09 | 0 | 0 | -0.039 | -0.035 | -0.034 | 0 | 0 | 0 | 0 | 0 | 0 | 0.105 | 0.331 | 0 | 0 | 0 | 0 |
| **PC2** | 0 | 0 | 0 | -0.101 | 0 | 0.031 | 0 | -0.105 | 0 | 0 | 0 | 0 | 0 | -0.016 | 0.105 | 0 | 0.225 | 0 | 0.085 | 0 | 0 |
| **PC3** | 0 | -0.029 | -0.054 | 0 | 0 | -0.036 | -0.04 | 0.061 | 0 | 0 | -0.056 | 0 | 0 | 0 | 0.331 | 0.225 | 0 | 0 | 0.045 | 0 | 0.025 |
| **ME1** | 0 | 0 | 0 | 0 | 0 | 0 | 0 | 0 | 0 | 0 | 0 | 0 | 0 | 0 | 0 | 0 | 0 | 0 | 0 | 0 | 0.066 |
| **ME2** | -0.045 | 0 | 0 | 0 | 0 | 0.072 | 0 | -0.207 | 0 | 0 | 0 | 0 | -0.031 | 0 | 0 | 0.085 | 0.045 | 0 | 0 | 0.129 | 0.134 |
| **ME3** | 0 | 0 | 0 | 0 | 0 | 0 | 0 | 0 | 0 | 0 | 0 | 0 | 0 | 0 | 0 | 0 | 0 | 0 | 0.129 | 0 | 0 |
| **ME4** | 0.12 | 0 | -0.049 | 0 | 0.032 | 0 | 0 | 0.043 | 0 | 0 | 0.037 | -0.033 | -0.025 | 0 | 0 | 0 | 0.025 | 0.066 | 0.134 | 0 | 0 |

**Table S1.** The weight-matrix of network among all patients

|  | **CP** | **PI1** | **PI2** | **PI3** | **IF1** | **IF2** | **IF3** | **IF4** | **MH1** | **MH2** | **AE1** | **AE2** | **AE3** | **AE4** | **PC1** | **PC2** | **PC3** | **ME1** | **ME2** | **ME3** | **ME4** |
| --- | --- | --- | --- | --- | --- | --- | --- | --- | --- | --- | --- | --- | --- | --- | --- | --- | --- | --- | --- | --- | --- |
| **CP** | 0 | 0 | 0 | 0 | 0.133 | -0.291 | 0 | 0.134 | 0 | 0 | 0 | 0.062 | 0 | 0 | 0 | 0 | 0 | 0 | -0.065 | 0 | 0.033 |
| **PI1** | 0 | 0 | 0.16 | 0.13 | 0.345 | 0 | 0.104 | 0 | 0 | 0 | 0 | 0 | 0 | 0 | -0.045 | 0 | -0.043 | 0 | 0 | 0 | 0 |
| **PI2** | 0 | 0.16 | 0 | 0.314 | 0 | 0 | 0.067 | 0 | 0.056 | 0 | 0 | 0 | 0 | 0 | -0.059 | 0 | -0.043 | 0 | 0 | 0 | 0 |
| **PI3** | 0 | 0.13 | 0.314 | 0 | 0 | 0.049 | 0.171 | 0 | 0.113 | 0 | 0 | 0 | 0 | 0 | -0.069 | -0.074 | 0 | 0 | 0 | 0 | 0 |
| **IF1** | 0.133 | 0.345 | 0 | 0 | 0 | 0.258 | 0.152 | -0.229 | 0 | 0.05 | 0 | 0.028 | 0 | 0 | 0 | 0 | 0 | 0 | 0 | 0 | 0.026 |
| **IF2** | -0.291 | 0 | 0 | 0.049 | 0.258 | 0 | 0 | 0 | 0 | 0 | 0.07 | 0 | 0 | 0 | 0 | 0 | 0 | 0 | 0.063 | 0 | 0 |
| **IF3** | 0 | 0.104 | 0.067 | 0.171 | 0.152 | 0 | 0 | 0 | 0.088 | 0.136 | 0 | 0 | 0 | 0 | 0 | 0 | -0.078 | 0 | 0 | 0 | 0 |
| **IF4** | 0.134 | 0 | 0 | 0 | -0.229 | 0 | 0 | 0 | 0 | 0 | 0 | 0.132 | 0 | 0 | -0.049 | -0.084 | 0.104 | 0 | -0.157 | 0 | 0 |
| **MH1** | 0 | 0 | 0.056 | 0.113 | 0 | 0 | 0.088 | 0 | 0 | 0.595 | 0 | 0.072 | 0 | 0 | -0.054 | 0 | 0 | 0 | 0 | 0 | 0 |
| **MH2** | 0 | 0 | 0 | 0 | 0.05 | 0 | 0.136 | 0 | 0.595 | 0 | 0 | 0.042 | 0 | 0.055 | 0 | 0 | -0.067 | 0 | 0 | 0 | 0 |
| **AE1** | 0 | 0 | 0 | 0 | 0 | 0.07 | 0 | 0 | 0 | 0 | 0 | 0.118 | 0 | 0.325 | 0 | 0 | -0.035 | 0 | 0 | 0 | 0 |
| **AE2** | 0.062 | 0 | 0 | 0 | 0.028 | 0 | 0 | 0.132 | 0.072 | 0.042 | 0.118 | 0 | 0.081 | 0.335 | 0 | 0 | 0 | 0 | 0 | 0 | 0 |
| **AE3** | 0 | 0 | 0 | 0 | 0 | 0 | 0 | 0 | 0 | 0 | 0 | 0.081 | 0 | 0.109 | 0 | 0 | 0 | 0 | 0 | 0 | 0 |
| **AE4** | 0 | 0 | 0 | 0 | 0 | 0 | 0 | 0 | 0 | 0.055 | 0.325 | 0.335 | 0.109 | 0 | 0 | 0 | 0 | 0 | 0 | 0 | 0 |
| **PC1** | 0 | -0.045 | -0.059 | -0.069 | 0 | 0 | 0 | -0.049 | -0.054 | 0 | 0 | 0 | 0 | 0 | 0 | 0.084 | 0.319 | 0 | 0 | 0 | 0 |
| **PC2** | 0 | 0 | 0 | -0.074 | 0 | 0 | 0 | -0.084 | 0 | 0 | 0 | 0 | 0 | 0 | 0.084 | 0 | 0.188 | 0 | 0.078 | 0 | 0 |
| **PC3** | 0 | -0.043 | -0.043 | 0 | 0 | 0 | -0.078 | 0.104 | 0 | -0.067 | -0.035 | 0 | 0 | 0 | 0.319 | 0.188 | 0 | 0 | 0 | 0 | 0.044 |
| **ME1** | 0 | 0 | 0 | 0 | 0 | 0 | 0 | 0 | 0 | 0 | 0 | 0 | 0 | 0 | 0 | 0 | 0 | 0 | 0 | 0 | 0 |
| **ME2** | -0.065 | 0 | 0 | 0 | 0 | 0.063 | 0 | -0.157 | 0 | 0 | 0 | 0 | 0 | 0 | 0 | 0.078 | 0 | 0 | 0 | 0.093 | 0.099 |
| **ME3** | 0 | 0 | 0 | 0 | 0 | 0 | 0 | 0 | 0 | 0 | 0 | 0 | 0 | 0 | 0 | 0 | 0 | 0 | 0.093 | 0 | 0 |
| **ME4** | 0.033 | 0 | 0 | 0 | 0.026 | 0 | 0 | 0 | 0 | 0 | 0 | 0 | 0 | 0 | 0 | 0 | 0.044 | 0 | 0.099 | 0 | 0 |

**Table S2**.The weight-matrix of network among male patient

|  | **CP** | **PI1** | **PI2** | **PI3** | **IF1** | **IF2** | **IF3** | **IF4** | **MH1** | **MH2** | **AE1** | **AE2** | **AE3** | **AE4** | **PC1** | **PC2** | **PC3** | **ME1** | **ME2** | **ME3** | **ME4** |
| --- | --- | --- | --- | --- | --- | --- | --- | --- | --- | --- | --- | --- | --- | --- | --- | --- | --- | --- | --- | --- | --- |
| **CP** | 0 | 0 | 0 | 0 | 0.097 | -0.321 | 0 | 0 | 0 | 0 | 0 | 0.027 | 0 | 0 | 0 | 0 | 0 | 0 | 0 | 0 | 0.156 |
| **PI1** | 0 | 0 | 0.176 | 0.173 | 0.319 | 0 | 0.078 | 0 | 0.06 | 0 | 0 | 0 | 0 | 0 | -0.019 | 0 | 0 | 0 | 0 | 0 | 0 |
| **PI2** | 0 | 0.176 | 0 | 0.322 | 0 | 0 | 0.149 | 0 | 0 | 0.08 | 0 | 0 | 0 | 0 | -0.041 | 0 | -0.062 | 0 | 0 | 0 | -0.044 |
| **PI3** | 0 | 0.173 | 0.322 | 0 | 0 | 0.068 | 0.12 | 0 | 0.079 | 0 | 0 | 0 | 0 | 0 | -0.103 | -0.091 | 0 | 0 | 0 | 0 | 0 |
| **IF1** | 0.097 | 0.319 | 0 | 0 | 0 | 0.226 | 0.187 | -0.152 | 0 | 0.083 | 0 | 0.042 | 0 | 0 | 0 | 0 | 0 | 0 | 0 | 0 | 0 |
| **IF2** | -0.321 | 0 | 0 | 0.068 | 0.226 | 0 | 0.116 | 0 | 0 | 0.051 | 0 | 0 | 0.044 | 0 | 0 | 0 | -0.073 | 0 | 0 | 0 | 0 |
| **IF3** | 0 | 0.078 | 0.149 | 0.12 | 0.187 | 0.116 | 0 | 0 | 0.113 | 0.073 | 0 | 0 | 0 | 0 | -0.058 | 0 | 0 | 0 | 0 | 0 | 0 |
| **IF4** | 0 | 0 | 0 | 0 | -0.152 | 0 | 0 | 0 | 0 | 0 | 0 | 0.087 | 0 | 0 | 0 | -0.067 | 0 | 0 | -0.199 | 0 | 0 |
| **MH1** | 0 | 0.06 | 0 | 0.079 | 0 | 0 | 0.113 | 0 | 0 | 0.592 | 0 | 0 | 0 | 0.045 | 0 | 0 | -0.036 | 0 | 0 | 0 | 0 |
| **MH2** | 0 | 0 | 0.08 | 0 | 0.083 | 0.051 | 0.073 | 0 | 0.592 | 0 | 0 | 0.105 | 0.054 | 0 | 0 | 0 | 0 | 0 | 0 | 0 | 0 |
| **AE1** | 0 | 0 | 0 | 0 | 0 | 0 | 0 | 0 | 0 | 0 | 0 | 0.094 | 0.077 | 0.356 | 0 | 0 | -0.052 | 0 | 0 | 0 | 0 |
| **AE2** | 0.027 | 0 | 0 | 0 | 0.042 | 0 | 0 | 0.087 | 0 | 0.105 | 0.094 | 0 | 0.118 | 0.301 | 0 | 0 | 0 | 0 | -0.042 | 0 | -0.027 |
| **AE3** | 0 | 0 | 0 | 0 | 0 | 0.044 | 0 | 0 | 0 | 0.054 | 0.077 | 0.118 | 0 | 0.053 | 0 | 0 | 0 | 0 | -0.026 | 0 | -0.025 |
| **AE4** | 0 | 0 | 0 | 0 | 0 | 0 | 0 | 0 | 0.045 | 0 | 0.356 | 0.301 | 0.053 | 0 | 0 | 0 | 0 | 0 | 0 | 0 | 0 |
| **PC1** | 0 | -0.019 | -0.041 | -0.103 | 0 | 0 | -0.058 | 0 | 0 | 0 | 0 | 0 | 0 | 0 | 0 | 0.101 | 0.33 | 0 | 0 | 0 | 0 |
| **PC2** | 0 | 0 | 0 | -0.091 | 0 | 0 | 0 | -0.067 | 0 | 0 | 0 | 0 | 0 | 0 | 0.101 | 0 | 0.229 | 0 | 0.063 | 0 | 0 |
| **PC3** | 0 | 0 | -0.062 | 0 | 0 | -0.073 | 0 | 0 | -0.036 | 0 | -0.052 | 0 | 0 | 0 | 0.33 | 0.229 | 0 | 0 | 0.049 | 0 | 0 |
| **ME1** | 0 | 0 | 0 | 0 | 0 | 0 | 0 | 0 | 0 | 0 | 0 | 0 | 0 | 0 | 0 | 0 | 0 | 0 | 0 | 0 | 0.085 |
| **ME2** | 0 | 0 | 0 | 0 | 0 | 0 | 0 | -0.199 | 0 | 0 | 0 | -0.042 | -0.026 | 0 | 0 | 0.063 | 0.049 | 0 | 0 | 0.137 | 0.128 |
| **ME3** | 0 | 0 | 0 | 0 | 0 | 0 | 0 | 0 | 0 | 0 | 0 | 0 | 0 | 0 | 0 | 0 | 0 | 0 | 0.137 | 0 | -0.034 |
| **ME4** | 0.156 | 0 | -0.044 | 0 | 0 | 0 | 0 | 0 | 0 | 0 | 0 | -0.027 | -0.025 | 0 | 0 | 0 | 0 | 0.085 | 0.128 | -0.034 | 0 |

**Table S3.** The weight-matrix of network among female patients

| Abbreviation | All | Male | Female |
| --- | --- | --- | --- |
| #CP | 1.498027416 | 1.777937036 | 0.830254346 |
| #ME1 | -0.852583264 | -0.928730004 | -0.62457563 |
| #ME2 | -0.443227856 | -0.716251132 | -0.45615444 |
| #ME3 | -0.706471157 | -0.688068373 | -0.581654333 |
| #ME4 | 0.278377937 | 0.012317324 | 0.101013386 |
| #PI1 | 1.234294185 | 0.752587234 | 1.465240315 |
| #PI2 | 0.081255193 | 0.35133291 | 0.243443627 |
| #PI3 | 0.211483558 | 0.507687522 | 0.323876209 |
| #IF1 | 0.971621287 | 1.043534143 | 1.102843724 |
| #IF2 | 0.093615422 | 0.184637227 | -0.268724173 |
| #IF3 | 0.699046497 | 0.723396923 | 1.041961052 |
| #IF4 | -2.28639643 | -1.827359129 | -2.368517124 |
| #MH1 | 1.117864199 | 1.32245118 | 1.226450261 |
| #MH2 | 1.489517501 | 1.168263588 | 1.671201176 |
| #AE1 | 0.695565929 | 0.620463685 | 0.312760616 |
| #AE2 | 0.801910148 | 0.825208668 | 0.713020235 |
| #AE3 | -0.575727129 | -0.701438109 | -0.40425219 |
| #AE4 | 0.924590937 | 1.200135337 | 0.987882021 |
| #PC1 | -0.648001147 | -0.603107281 | -0.324544672 |
| #PC2 | -0.331608288 | -0.432204723 | -0.262649759 |
| #PC3 | -0.365224344 | -0.503087854 | -0.235944206 |

**Table S4.** Expected Influence (*EI*) in the network of all, male and female patients.


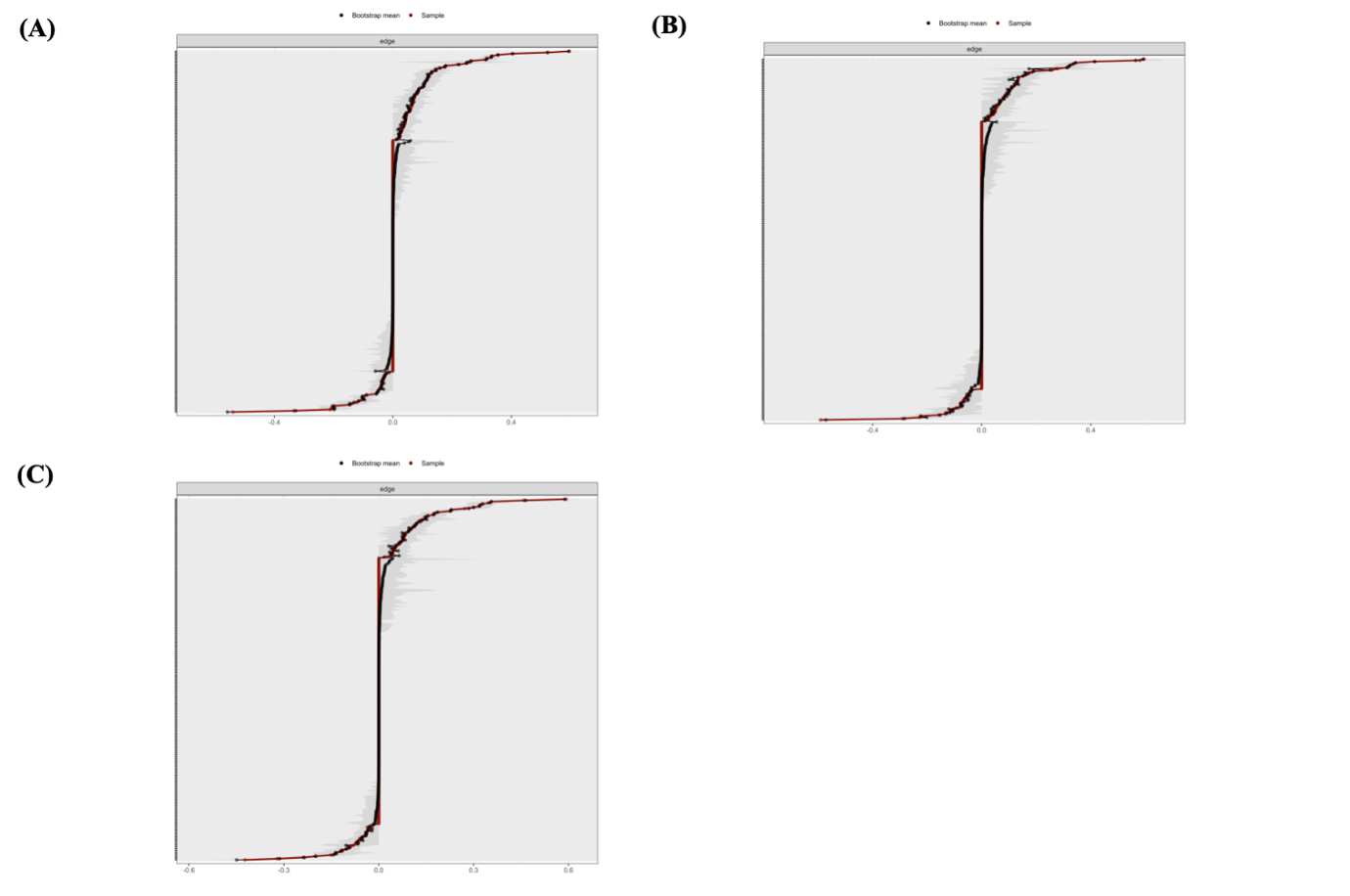


**Fig. S1**. Nonparametric bootstrapped confidence intervals of estimated edges in the network of all, male and female patients. The red line represents the estimated edge, while the dark area indicates the 95% bootstrap confidence interval


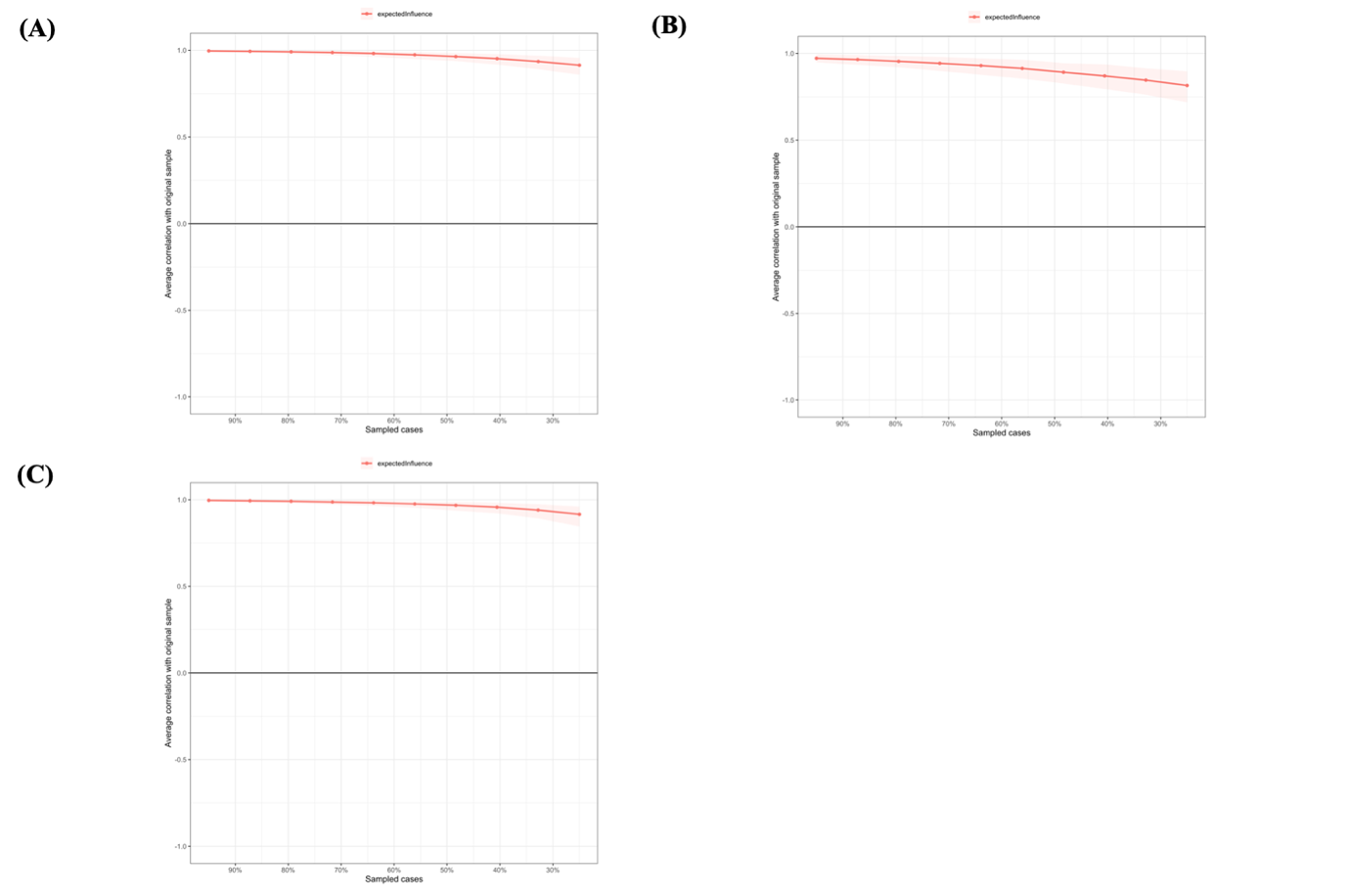


**Fig. S2**. The stability of centrality indices (*EI*) using case-dropping bootstrap in the network of all, male and female patients. The x-axis indicates the percentage of cases of the original sample included at each step. The y-axis indicates the average correlations between the original network's *EI* and the *EI* from the networks that were re-estimated after excluding increasing percentages of cases.

|  | Unit converted from | Recommended OME conversion factor (to convert to mg/day) |
| --- | --- | --- |
| Buprenorphine | mg/day | 75 (IV/IM) |
| Codeine | mg/day | 0.1 (PO)  0.3 (SC/IV) |
| Fentanyl | mg/day | 0.2 (IV/IM/SC) |
| Hydrocodone | mg/day | 1.2 (PO) |
| Hydromorphone | mg/day | 5.0 (PO)  17.5 (SC/IV) |
| Morphine | mg/day | 1. (PO)   3.0 (SC/IV) |
| Oxycodone | mg/day | 1.5 (PO)  3.0 (SC/IV) |
| Meperidine | mg/day | 0.1 (PO) |
| Pethidine | mg/day | 0.4 (IV/IM) |
| Tapentadol | mg/day | 0.4 (PO) |
| Tramadol | mg/day | 0.2 (PO) |
| Alfentanil |  | 30 (IV) |
| Sufentanil | mcg/day | 0.5 (BUC/SL)  2.0 (SC/IV) |
| Nalbuphine | mg/day | 3.0 (IV) |
| Levorphanol | mg/day | 13.8 (PO) |
| Meperidine | mg/day | - 1. (PO) |
| * IV: Intravenous, IM: Intramuscular, SC: Subcutaneous, PO: Oral, BUC: Buccal, SL: Sublingual; Please note that opioid analgesics without studied OMEs conversion factors were excluded. | | |

**Table S5.** Recommended Oral Morphine Equivalents (OMEs) conversion factors derived from existing literature and published studies
